# Celsius: a community resource for Affymetrix microarray data

**DOI:** 10.1186/gb-2007-8-6-r112

**Published:** 2007-06-14

**Authors:** Allen Day, Marc RJ Carlson, Jun Dong, Brian D O'Connor, Stanley F Nelson

**Affiliations:** 1 Department of Human Genetics, David Geffen School of Medicine, University of California, Los Angeles, California, 90095, USA

## Abstract

Celsius is a data warehousing system to aggregate Affymetrix CEL files and associated metadata. It provides mechanisms for importing, storing, querying, and exporting large volumes of primary and pre-processed microarray data. Celsius contains ten billion assay measurements and affiliated metadata. It is the largest publicly available source of Affymetrix microarray data, and through sheer volume it allows a sophisticated, broad view of transcription that has not previously been possible.

## Abstract 2

Celsius is a new system that serves as a warehouse by aggregating Affymetrix files and associated metadata, and containing the largest publicly available source of Affymetrix microarray data.

## Background

DNA microarrays have become the most important source of experimental genomic information that are applied in a large scale. They are widely used for tissue/disease classification as well as gene function discovery. Applications of this technology are routinely and widely published within almost all aspects of biology and human disease studies, with more than 14,000 PubMed citations containing the word 'microarray' published between 1996 and 2007. Even in the early years of microarray experimentation, it was widely recognized that a central repository of this information should be created to house these data. This enables potentially important additional information to be gleaned by re-interpretation by other researchers, perhaps in different contexts or in relation to new data. Thus, major efforts to house such data were made, namely the Gene Expression Omnibus (GEO) [1] and ArrayExpress (AEX) [2]. These repositories contain more than 82,000 and 50,000 microarray hybridizations of data, respectively. Primary data are expensive and time consuming to generate. In spite of the high cost, such experiments are rarely fully mined for their information content. Indeed, several meta-analyses have been reported that were based on archived data [3,4]. These studies demonstrate the benefit of data repositories and that additional inferences are possible with reanalysis.

Although gene expression microarray technology has been implemented in a variety of formats (spotted cDNAs, spotted column-synthesized oligos, and *in situ *synthesized oligos), the leading commercial supplier of microarrays has been Affymetrix Inc. (Santa Clara, CA, USA) since 1996. Within the GEO repository Affymetrix platforms account for 35% of all arrays deposited, but they represent approximately 60% of the genome-scale gene expression data. For instance, Affymetrix platform arrays account for the top seven array platforms in terms of the number of arrays deposited in GEO. Thus, in the public domain within repositories, this platform type forms the richest set of expression information that can be most readily combined in a useful manner for meta-analyses spanning multiple experiments. Furthermore, the Affymetrix platform has a standard set of protocols for probe generation and labeling, uses a single color detection system, and has a relatively reliable array fabrication process. The Affymetrix platform is widely applied to a variety of biologic problems. Thus, this platform is highly attractive as the basis for amalgamation of data from many different sources. In theory, historic arrays can be directly compared with additional experiments and provide an important tool for comparative analyses. However, because of the large number of analytical procedures for normalization and quantification from the oligonucleotide level data, it is greatly preferable to reanalyze primary data in the form of processed image files (termed CEL files, or CELs). This permits substantially more robust comparisons between datasets because the same analytical metric can be applied to the joint data and will ultimately permit more thorough vetting of algorithms to assess gene expression levels from this platform.

**Figure 1:**
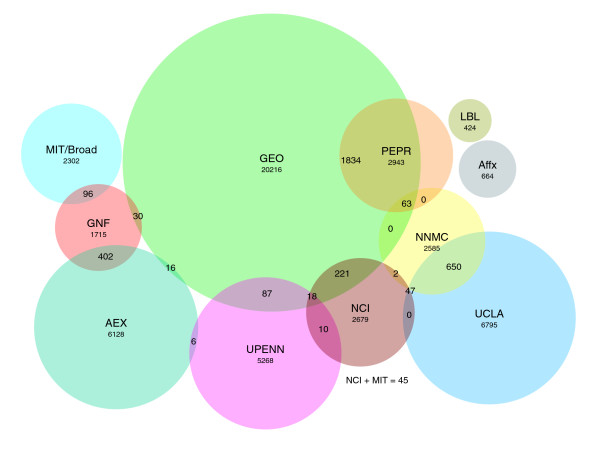
Summary of data sources present in Celsius. Data have been imported from several sources, 11 of which are shown. Numerals indicate the number of files within each source. Circle overlap is proportional to CEL overlap between data sources. AEX, EBI ArrayExpress [49]; AFFX, Affymetrix [50]; GEO, NCBI Gene Expression Omnibus [51]; GNF, Genomics Institute of the Novartis Research Foundation [52]; LBL, Lawrence Livermore National Laboratory; MIT, Broad Institute [53]; NNMC, NIH Neuroscience Microarray Consortium [54]; PEPR, Public Expression Profiling Resource [55]; UCLA, University of California, Los Angeles DNA Microarray Core Facility [56]; UPENN, University of Pennsylvania Microarray Core Facility [57].

Based on the popularity and ease of use of the Affymetrix platform we began to construct a combined resource for the storage of publicly available CELs for ongoing comparison with data generated at the University of California, Los Angeles (UCLA) DNA Microarray Core Facility as part of the National Institutes of Health Neuroscience Microarray Consortium (NNMC). The purpose of this assembly of CELs was to create a substantial reference set of primary data that would then be available for all ongoing projects. As we examined the available CEL file resources, it became apparent that fragmentation of public data into multiple small repositories has effectively occurred despite the presence of two major repository efforts and deposition requirements of journals. Of the more than 30,000 instances of CELs that were collected from 11 institutional servers (Figure 1) [1,2,5], fewer than 5% are present as CELs in either GEO or AEX, the two official public repositories. We estimate that up to 90% of generated CELs are not yet deposited in AEX or GEO. In fact, most public CELs are not easy to find. This suggests that the number of publicly available CELs is much larger than that used in our study, but these CELs are not accessible using standard bulk-mode data retrieval network protocols such as network file transfer protocols FTP and Rsync.

We further note that inconsistent annotation of experiments impedes meta-analysis. Re-use of these data is compromised by the low quality of clinically or experimentally relevant annotated metadata actually available for many datasets, as well as the inconsistent and incomplete implementation of the standards for encoding these metadata [6,7]. For instance, no repository uses controlled vocabularies, and therefore the annotation of experiments can be ambiguous and difficult to use when integrating datasets.

Here, we present a community-oriented structure to permit massive amalgamation of microarray data for joint analyses. We have termed this resource 'Celsius', to reflect both the intended community spirit and the restriction to image files generated from the Affymetrix platform. Celsius has four major goals: to import all available Affymetrix primary data, whether published or not, specifically gene expression, genotyping, and tiling CELs; to process imported data using best-of-breed statistical methods made available by the community; to facilitate and encourage community involvement in annotation of deposited samples using controlled vocabularies; and to make available for re-export consistently quantified and normalized data that can be combined without further processing. In this article we describe the methods employed to create this resource, a snapshot of its contents, nascent systematic approaches to annotate samples and genes solely using expression data, and growth rate.

## Results and discussion

### Data overview

**Figure 2:**
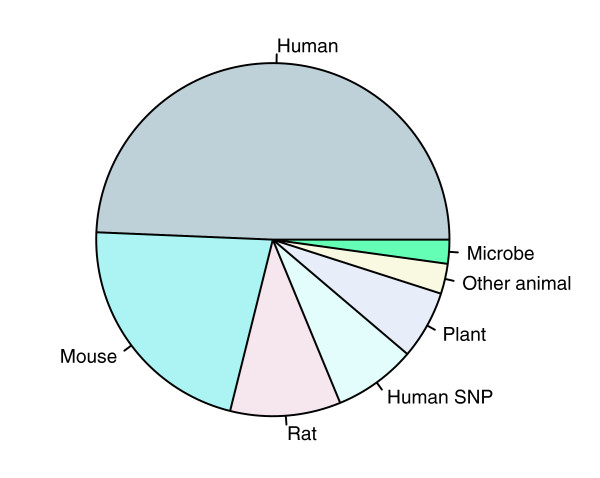
Tally of CELs by organism as of January 2007. SNP, single nucleotide polymorphism.

Celsius contains an agglomeration of more than 61,000 CEL files, each of which represents a single microarray hybridization performed using Affymetrix technology on one of 156 different array designs. The majority (67%) of CELs are derived from only ten array designs, as shown in Table 1. Of all CELs in Celsius, 95% contain gene expression measurements, 4% contain human DNA allelic copy number measurements, and the remainder of the CELs contain tiling and re-sequencing data. Within the gene expression data, nearly 50% were collected from human tissues or cell lines and nearly 20% from mouse tissues or cell lines (Figure 2).

**Table 1:** Most common Affymetrix array designs represented in Celsius

Platform	Number of CELs	Percentage of CELs	Organism
HG-U133A	11,296	19%	Human
HG-U133 Plus 2	5,954	10%	Human
HG U95Av2	4,600	8%	Human
ATH1-121501	3,952	6%	Plant
MG U74Av2	3,840	6%	Mouse
Mouse430 2	3,580	5%	Mouse
MOE430A	3,154	5%	Mouse
HG-U133B	2,522	4%	Human
RG U34A	2,258	4%	Rat
RAE230A	1,247	2%	Rat
Total	42,403	70%	

**Figure 3:**
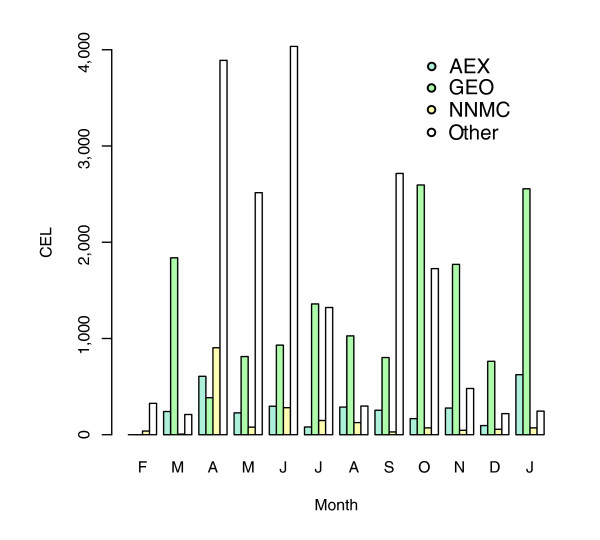
Monthly tally of CEL file import into Celsius from February 2006 to January 2007. AEX, EBI ArrayExpress; GEO, NCBI Gene Expression Omnibus; NNMC, NIH Neuroscience Microarray Consortium.

**Figure 4:**
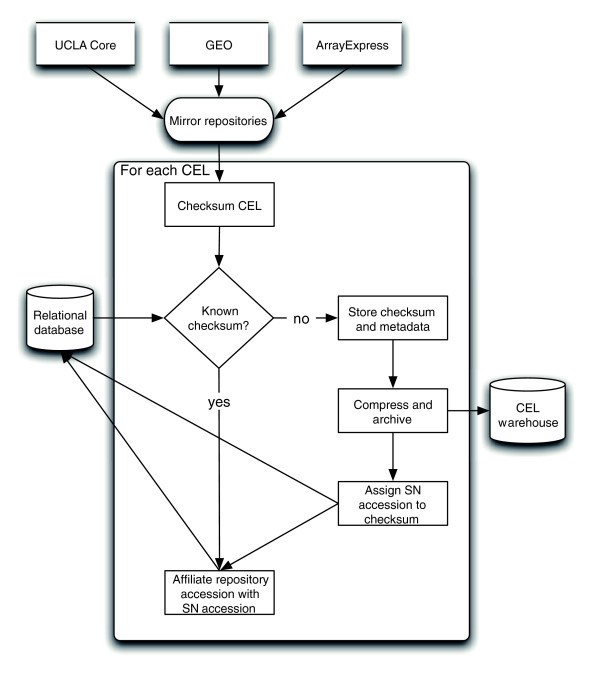
Process for importing microarray data from other repositories. Potentially novel CELs are checksummed and associated with a Celsius serial number database identifier (SNID) database accession identifier. Metadata from the source repository (sample accession, dataset accession), as well as metadata from the CEL (checksum, array type), are archived to a relational database. If a CEL not currently present in Celsius is detected, a then a SNID is assigned and the CEL is compressed and archived. Quantification is performed and resulting data are stored in a relational database. GEO, NCBI Gene Expression Omnibus; SN, University of California, Los Angeles DNA Microarray Core Facility.

Only primary data are imported, all of which were collected using the Affymetrix platform, a popular technology that represents more than 70% of all microarray data in GEO. The primary data in Celsius are the union of CELs collected from more than 11 institutions, including the two central repositories for microarray data: GEO and AEX. Celsius is continuously updated, and has a growth rate of 1,000 CELs/week, as observed from January 2006 to January 2007 (Figure 3). The size and growth rate of this dataset are corrected for inter-repository as well as intra-repository file-level redundancy, because approximately 5% of CELs are available from more than one institution or are available as replicates but by multiple database accession identifiers from a single institution. As of January 2007, Celsius is the world's largest publicly accessible resource for microarray data derived from the Affymetrix platform and contains three times as many CELs as GEO and ten times as many CELs as AEX, which are the largest public microarray data repositories. An illustration of the CEL load process is given in Figure 4. This illustrates redundancy checking and assignment of the serial number database identifiers (SNIDs; the primary database accession identifiers used by Celsius).

### Data processing

CELs loaded into the Celsius system are processed using many best-of-breed statistical quantification algorithms, including dChip, BRLMM, GC-RMA, MAS5, PLIER, RMA, and VSN [8-12]. Some of these algorithms are in the multi-array class of algorithms and require co-processing a batch of CELs to provide a confident signal estimate for each of the probesets. Each CEL loaded into the Celsius system is processed together with a selected 'quantification pool' of 50 CELs of the same array design that is held constant for all quantification events. We chose this method based on our observation that a quantification pool of this size is sufficient for all algorithms to estimate a signal stably, provided the pool was created from a heterogeneous mixture of samples. Corroborating findings using a similar approach were recently reported [13]. This 'quantification pool' technique allows Celsius to grow the dataset incrementally while ensuring that quantified values from each CEL are compatible for analysis with values from all other CELs. The code for managing CEL quantification is modular so that as new algorithms become available for processing microarray data extensions to the Celsius quantification pipeline can be readily implemented.

### Data access

All contents of Celsius may be accessed through use of the Celsius software library written in the R statistical programming language. It may be downloaded as the Celsius from the Comprehensive R Archive Network [14]. The Celsius library provides an application programmer interface (API) to a subset of the Celsius web services for both reading and write data, and is designed for seamless operation with components of the Bioconductor project [15,16]. Specific instructions for how to obtain, install, and use this library are provided at the Celsius project homepage [17]. We chose to focus on opening public access to Celsius through a programmatic API written in R because it is the *de facto *standard environment for the analysis of microarray data.

### Experimental metadata and community participation

The Celsius policy of microarray data sharing is liberal and inclusive when contrasted with the status quo. Rather than adhering to the Minimal Information About a Microarray Experiment [18] guidelines recommendation on data deposition (namely, that metadata for an experiment be provided concomitantly with primary data submission to a repository), we instead adopt the successful data sharing model of the International Nucleotide Sequence Database Collaboration (INSDC) [19]. In the INSDC model, primary data can be contributed to a public repository with or without metadata.

In contrast to other web-accessible microarray resources, additional metadata can be subsequently provided to the repository by anyone, not only by the contributor of the primary data. Celsius places strong emphasis on community participation. This is most evident in the system's ability to accept community contributions in the form of primary data as well as metadata. Indeed, public users of the Celsius system are able to upload, either anonymously or with attribution, primary data in the form of CEL files. These are processed as all other CELs in the system; they are archived, quantified, and then made publicly visible and annotatable.

Users may annotate all SNIDs and probeset records present in Celsius, either through the use of ontology terms approved by the National Center for Biomedical Ontology (NCBO) [20], such as the Gene Ontology (GO) and Mouse Anatomy Ontology [21,22], or by using free-text 'tags'. These activities are possible through programmatic web service APIs (described below under Web services). Likewise, records for CELs and probesets may be retrieved from Celsius using ontology identifiers. We created these interfaces to allow the community to import and export data and metadata as easily as possible and in a distributed manner. Our aim in creating these interfaces is to create a metadata resource with broad coverage that permits analysis over an integrated set of data produced through the efforts of the community.

**Figure 5:**
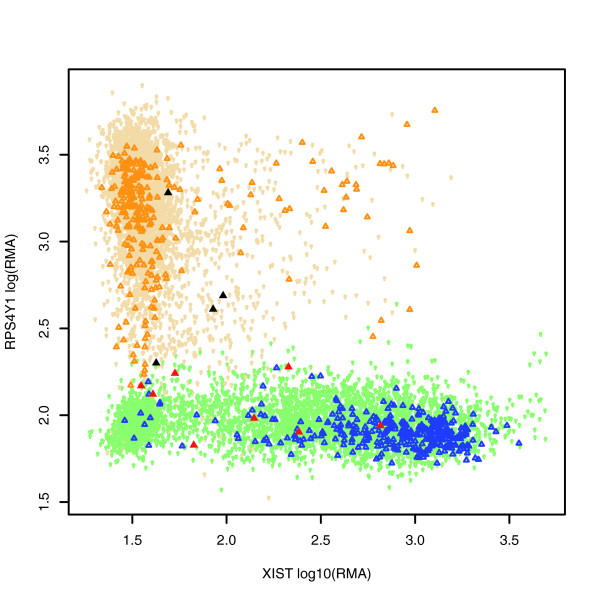
Human HG-U133A CELs are automatically classified for sex of the tissue or cell line of origin. Orange points are manually curated as male and are also correctly classified as male. Red points are manually curated male that are falsely classified as female. Wheat points are classified as male but do not have manually curated results. These three types of points are also denoted by different shapes in the order of triangle, filled triangle, and circle respectively. All points are classified by assigning two clusters in five-dimensional probeset space, two of which are shown. x-axis, 221728_x_at, *XIST*; y-axis, 201909_at, *RPS4Y1*.

The community annotation features of Celsius have already been used to manually encode annotation for more than 30% of all HG-U133A CELs (Figure 5a). This number continues to grow as driven by user demand, both inside and outside our group. These CELs are annotated for tissue of origin, cell type of origin, pathologic state, or phenotypic state of the hybridized biologic sample. The current state of annotation for HG-U133A CELs for tissue and neoplastic pathologic state is shown in Figure 5b,c. After careful review of the available descriptions of experiment design and sample treatment, these annotations were manually encoded using controlled vocabularies provided by public ontology efforts [22-26]. The process of encoding annotation with controlled vocabularies is difficult and time consuming, because it frequently requires review of the primary literature to obtain key facts about the biologic samples. Our intention is that the community annotation features will promote distribution of the effort to annotate CEL files gathered into Celsius, both by manual curation and by programmatic extraction of annotation from literature and GEO/AEX annotation deposition.

The past few decades have shown that, in general, this policy of open and minimal participation is beneficial. By allowing primary data deposition even without any metadata, and *vice versa*, a flood of primary data has entered public sequence repositories. This condition fostered the growth of a large and active sequence analysis research community. We believe the INSDC data sharing policy can be successfully applied to data and metadata derived from all high-throughput genome-scale assays. We demonstrate the application of this policy in Celsius.

### Web services

To facilitate the sharing of data from Celsius, a series of programmatic interfaces have been developed following the web services model of information exchange. This design is attractive because of its platform neutrality, so researchers can interact with the Celsius services using a wide variety of programming languages. XML (extensible markup language) and web-based protocols were used to facilitate this ease of access to Celsius for researchers embracing large-scale microarray experimentation and data analysis through bulk data access.

The services available though Celsius provide a wide range of abilities to query, transform, and upload microarray data. For example, Celsius web services provide an identifier transformation service that allows researches to query the system with one database accessor and retrieve all others with which a given sample is associated. This allows the mapping of a GEO identifier to an AEX identifier via a SNID identifier intermediate, checking whether a given CEL is present, and, perhaps in the future, automatic import of experimental metadata. Given the distributed nature of microarray datasets, and the possibility of the same dataset being present in multiple repositories, this accession transformation service is an invaluable resource for the microarray community.

Several query web services complement the look-up services and provide a mechanism for searching experimental metadata within Celsius. These services take advantage of the extensive use of ontology annotations throughout Celsius and allow for the rapid identification of all annotated SNIDs of a particular type and level of certainty. For instance, a query for manually curated nervous tissue uses the structure of the controlled vocabulary to return not just SNIDs annotated as nervous tissue, but also all SNIDs annotated with controlled vocabulary terms that are part of the nervous system, such as spinal cord. Other search services include identification of samples based on platform, data retrieval by normalization algorithms, and searching of free-text tags.

Exemplifying the inclusive position of Celsius toward microarray data, we provide a deposition service that can be used either anonymously or with attribution. This function permanently archives CEL data, and all uploaded CELs are assigned a SNID and quantified. They can subsequently be retrieved in SOFT format and submitted to GEO, thus meeting current journal data deposition requirements. This upload service is complemented by a curatorial service that allows Celsius contributors to attach both ontology-based and free text annotations to any sample records.

Other web services are also available from Celsius and more will be added in the future. Up-to-date documentation of what web services are available can be found at the Celsius project homepage [17]. Data from each service are available in both XML and tabular formats so that they may easily be imported into many programming environments.

### Annotation examples

The Celsius community features have been used programmatically to annotate a large number of samples and genes. We present two examples to demonstrate the wealth of information that can be gleaned from a data resource of this magnitude. Automated annotation algorithms such as these will become increasingly common in Celsius, similar to the way vector trimming and gene predictions algorithms are routinely run on nucleotide data as they are produced by sequencers.

#### Assignment of sex annotation to CELs

**Figure 6:**
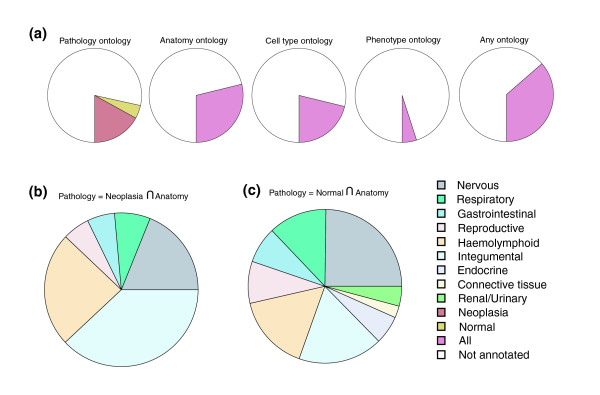
Annotation coverage and depth for the Human HG-U133 platforms. **(a) **Filled wedges indicate the fraction of CELs for which annotation is present. The red and yellow wedges of the left-most pie indicate fraction of diseased and normal samples, respectively. The right-most pie's wedge indicates the fraction of CELs for any annotation from the preceding columns have been given (excluding sex). **(b) **Human HG-U133A samples grouped by tumor type and normal. Annotation was manually assigned after literature review. Many integumental system tumors are breast tumors. **(c) **Human HG-U133A samples grouped by tissue of origin. Annotation was manually assigned after literature review.

We assigned each of the 8,915 HG-U133A SNIDs present in Celsius as of October 2006 into male/female classes. This was achieved using the R package mclust's Mclust function with default options to assign points into two clusters. Cluster assignment was based on RMA processed gene expression values of two probesets on the X chromosome and three probesets on the Y chromosome (Figure 6). Of these 8,915 SNIDs, 624 were previously manually assigned to one of the male/female classes using external information, which we regard as accurate. Details for retrieving these data are described below under Materials and methods.

Table 2 shows the fraction of correctly and incorrectly assigned male/female labels by the clustering method. For the female class, the false-positive rate is 8/349 = 0.0229 and the false-negative rate is 8/279 = 0.0287. For the male class, these rates are 4/275 = 0.0145 and 4/345 = 0.0116, respectively. The rand index and rand index corrected for agreement by chance [27,28] for Table 2 are 0.9622 and 0.9244, respectively. The male class has a lower false-positive and a higher false-negative rate. This is probably due to the strong dependence of female classification on *XIST *expression, which is typically high in all female derived cell lines and tissues but can be down regulated in some disease states. Overall, the classification method based on gene expression data works very well in assigning sex class, and it enables large-scale analyses based on sex that were not previously possible using the manually encoded annotations, even though a small error rate is added.

**Table 2:** Assignment of sex-annotated HG-U133A SNIDs by clustering

	Curated female	Curated male	Total
Classified female	341	8	349
Classified male	4	271	275
Total	345	279	624

SNID, serial number database identifier.

#### Assignment of Gene Ontology biological process annotation to probesets

**Figure 7:**
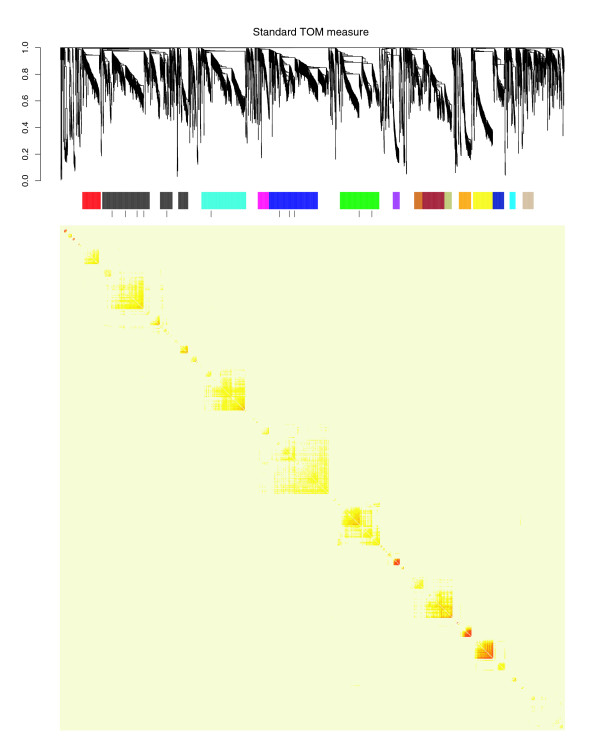
A gene network constructed from 3600 most varying human probesets. The hierarchical clustering tree and the heat map of the topologic overlap matrix for the 3600 HG-U133A probesets with the largest coefficients of variation measured across 1078 HG-U133A serial number database identifiers (SNIDs) that were annotated as pathologically normal. The color breaks in the colored annotation bar above the heat map mark annotation groups of probesets based on EASE, and tick marks mark the individual modules of highly interconnected probes before being merged into a single annotation group. Colors, left to right are defined as follows: red, transcription; black, response to biotic stimulus; turquoise, ectoderm development; magenta, regulation of metabolism; blue, nervous system development; green, muscle contraction; dark orchid, digestion; chocolate, organic acid metabolism; brown, acute-phase response; dark khaki, complement activation; orange, pregnancy; yellow, sexual reproduction; midnight blue, mitotic cell cycle; deep sky blue, skeletal development; tan, phosphate transport.

Genes with similar expression patterns are thought to be more likely to be functionally associated [29]. They may form structural complexes, participate in the same biochemical pathway, or be regulated by a common transcriptional mechanism. Gene co-expression networks are constructed on the basis of microarray data from the transcriptional response of cells to changing conditions [30,31]. In these networks a node corresponds to an individual probeset-based measurement of a given gene. We constructed such a network of 3,600 probesets with the greatest coefficients of variation measured across 1078 HG-U133A SNIDs that were annotated as pathologically normal using previously described methods [31,32]. We identified 35 modules within this network that correspond to well separated branches of the resulting hierarchical clustering tree. They are visualized as blocks along the diagonal of the topologic overlap matrix (TOM), as shown in Figure 7. The TOM measure uses the neighbor information instead of just their direct connection strength (adjacency) and is thus a robust measure of interconnectedness. This is similar to a gene cluster. More details about the topologic overlap measure, along with a tutorial using freely available R software to construct gene co-expression networks and to identify modules, can be found in the Materials and methods section, below. The parameters and other settings specifically used in this application are listed there for readers to replicate this analysis.

Modules in Figure 7 are color coded by the most significantly enriched GO biologic process (BP) [21] as computed with EASE [33]. Modules that did not have any significantly enriched BP (Bonferroni *P *value > 0.05) were not considered for further analysis (*n *= 5). Many of the remaining 30 modules shared common BP and were merged, leaving 15 distinct annotation groups. All 15 of these groups are shown in Figure 7. The green group of probesets (*n *= 282) is enriched for probesets involved in muscle contraction (*P *= 2.33 × e^-42^). Of the 188 probesets in this group that are annotated for any BP, 59 (31%) were previously known to be involved in muscle contraction, which correspond to 68% of the 87 probesets contained in the analyzed population of 3,600 probesets that are associated with muscle contraction. The green group corresponds to a bright block along the diagonal of the TOM plot. It indicates that the probesets within this group have high topologic overlap measures as well as highly correlated expression profiles.

Use of the primary BP assigned by EASE to annotate uncharacterized or partially characterized probesets warrants further exploration, but these data cannot be used for classification using conventional methods.

This is because gene annotation is incomplete, and it is therefore not possible to estimate the false-positive rate in assigning an annotation to a probeset. However, the 223 probesets within the green module not known to participate in muscle-specific processes are highly correlated with probesets known to be involved in these processes, and therefore they may play a role in muscle tissue. Numerous other gene-gene correlations provide additional information about the specific expression of genes within specific tissue types.

## Conclusion

Celsius is a substantial data resource that contains more primary and derivative microarray measurements than all public repositories combined. Celsius was assembled and continues to grow by means of permissively importing Affymetrix CEL files and assigning SNID database accession identifiers to them. Initially, data from 11 independent institutions were imported. Celsius continues to add more institutions to this list and imports data from all available institutions on a weekly basis. Imported data are processed using best-of-breed signal estimation algorithms. Metadata are acquired and associated with SNIDs through both manual and automated curatorial processes. Access to all contained data and metadata are provided to the public through easy-to-use programmatic interfaces, along with online documentation and prefabricated software libraries for data extraction. Celsius is a useful amalgamation of primary data for development and testing of quantification algorithms, individual probe level analyses, and identification of gene-gene relationships and gene networks; furthermore, it provides reference material for ongoing work using these array platforms. We encourage further enhancement of this dataset by the community through its programmatic and manual interfaces for upload of primary data and metadata. We continue to stimulate the growth of an active and collaborative environment for the development of gene expression inference algorithms, similar to that created by the establishment of large nucleic and peptide sequence databases. By assembling, redistributing, and creating mechanisms for large-scale community involvement in working with these data, a turning point will be reached such that high-throughput genomic data will be reused, mined, and analyzed to its full potential.

## Materials and methods

### Data import

Initially, all public CELs identifiable at AEX and GEO were copied to UCLA by FTP mirror. This was performed in January 2006 in bulk. Subsequently, additional data sources were added as institutions have been willing to permit upload into Celsius. Since the initial data upload in bulk, additional data from these sources are automatically mirrored on a weekly basis (Figure 4). The data import process begins by creating a local mirror for each of the sites from which data will be imported. The contents of these repository mirrors are then scanned for all CELs present. For each CEL, an MD5 checksum is calculated and associated with the CEL's accession from the remote data source (for instance, a CEL from GEO is associated with a GSM [GEO sample] accession). Most new data from this mirroring process derive from AEX, GEO, the UCLA DNA Microarray Facility, and NNMC. When Celsius detects a CEL that has not previously been imported, file-level metadata (file format, platform, and checksum) are extracted, a permanent SN database accession identifier is assigned, and the file is compressed and permanently stored on an archival file system. The assigned SN database accession identifier (SNID) can be used by both internal and external applications to refer to that CEL's data in Celsius. Unique CELs are identified using a checksum algorithm. This is necessary because a single CEL may exist in multiple repositories but under a different name at each repository. Finally, each unique CEL is processed on a 16-node cluster of computers administered using Sun Grid Engine. We use several common quantification algorithms, namely dChip, gcRMA, RMA, PLIER, MAS5, and VSN [8-11]. These algorithms are available from the Bioconductor suite of bioinformatics utilities [34]. Quantified expression values for each probeset from each CEL are produced by processing it along with a platform-specific pool of 50 other CELs, where the pool is held constant for each CEL that is processed. A similar procedure was recently described and validated [13].

### Programmatic access

All data in Celsius can be accessed using the Celsius R library. The package itself and instructions on its use are available at the Celsius project homepage [17]. This service utilizes an extension to the DAS2 (Distributed Annotation System 2.0) protocol [35,36], which allows assay data to be provided as hyperlinked Microarray Gene Expression Markup Language (MAGE-ML) fragments. In addition to the DAS2 service, other Celsius-specific services also documented at the homepage [17] are also available. Notable among these are the following: an identifier transformation service for mapping external database accession identifiers such as the GEO GSM sample identifier to and from Celsius SNIDs; a matrix label generation service, which can be used to create textual descriptions suitable as sample descriptors, for instance as row/column labels in a heatmap; a curatorial service that enables users to contribute to Celsius through attaching ontology and free-text metadata to existing CEL and probeset records; and a CEL deposition service, which allows primary data to be deposited anonymously and can be subsequently extracted in SOFT format suitable for upload to GEO.

Open source libraries for interacting with the Celsius web services have been written in the R and Java computer programming languages. These libraries are available from the Comprehensive R Archive Network [14] and Genoviz websites [14,37,38].

### Data representation

At its core, Celsius is a relational data warehouse based on PostgreSQL [39] and is designed for online analytical processing. Given the scale of data to be stored, the ability to respond to user queries in minimal time has been a major design goal in all aspects of system design. Celsius is implemented using the Chado database schema, which is a component of the Generic Model Organism Database Project [40]. The MAGE module of the Chado schema that is pertinent to the representation and storage of microarray data is presented in Additional data file 1. The schema has been optimized to accommodate several classes of user requests: to retrieve signal estimates calculated with algorithm A for all probesets on quantified CEL Q; to retrieve signal estimates calculated with algorithm A for all CELs on probeset P; to calculate distance from signature p to all samples using metric D and signal estimates calculated with algorithm A; to calculate distance from signature q to all probesets using metric D and signal estimates calculated with algorithm A; to retrieve all annotations on CEL Q; to retrieve all CELs annotated at or below ontology term T; and to annotate CEL Q with term T.

If implemented as a single table, it is impossible to simultaneously minimize query time for both cases 1 and 2. Minimization is achieved through clustering, or physically ordering disk blocks by an index, and it is not possible to have more than one physical ordering for a table. Thus, minimizing query time for case 1 necessarily increases query time for case 2. Optimization of query time for cases 3 and 4 presents the same problem because these cases are dependent upon cases 1 and 2. We overcame this obstacle by storing identical data in two tables and clustering each table on a different index. By using this technique, the size of the tables containing signal estimates is doubled. However, the advantage is that the retrieval time is reduced by several orders of magnitude by lessening hard disk activity. We have also chosen to partition the table that holds all signal estimates based on quantification algorithm, denoted A. This optimization reduces the number of rows in each table and results in a proportional query time of log2(n/N), where n is the number of CELs processed with algorithm A and N is the number of CELs multiplied by the number of quantification algorithms used.

Typical functions to be performed on Celsius involve matrix manipulations using the R programming language. Although it is possible to perform these calculations through a call to an external instance of R, it is inefficient. We make use of PL/R [41], a procedural extension to the PostgreSQL database that allows an embedded R instance to run inside the PostgreSQL environment. This technique allows R functions to be called as part of a standard structured query language (SQL) query. For instance, the calculation of correlation coefficients for all pairs of probesets from a particular array design can be performed. This method is used to infer gene interaction networks [30] from gene expression data, which otherwise is very costly to perform.

Cases 4 through 7 can be accommodated for a single user's ontology-based annotations by using the stock Chado schema for representing CELs, their biologic source materials, and associated annotations. We extended the schema to support storage of annotations from multiple users through the creation of a user module that associates all annotations with a particular user. We also added the ability to both attach and search free-text 'tags' using PostgreSQL's Tsearch2 extension [42].

#### Sex annotation

We assigned each of the 8915 HG-U133A SNIDs present in Celsius as of October 2006 into male/female classes using the R mclust package's Mclust function with default options to assign points into two clusters. Cluster assignment was based on RMA processed gene expression values of five probesets: 214218_s_at and 221728_x_at on chromosome X and 201909_at, 206769_at and 205000_at on chromosome Y. Of these 8,915 SNIDs, 624 SNIDs were previously manually assigned to one of the male/female classes using external information, which we regard as accurate. All HG-U133A samples may be retrieved from Celsius by using the Celsius R library referenced in the section Programmatic access (above) and retrieving all HG-U133A measurements for these five probesets.

#### Gene coexpression network construction

Using previously described methods [31], we calculated the Pearson correlation matrix for the gene expression profiles of the 3,600 probesets across 1078 HG-U133A SNIDs annotated as pathologically normal. To reproduce these results, these data may be retrieved from Celsius using the following R commands after installing the Celsius R library referenced in the section Programmatic access and searching for HG-U133A samples matching the 'normal' (MPATH:458) ontology term.

We raised all elements in the matrix to the power 6 to create the adjacency matrix. The adjacency matrix is equivalent to an undirected weighted network. Using the topologic overlap measure [32], we calculated a TOM from the adjacency matrix. The topologic overlap measure between two nodes is approximately the proportion of the number of shared neighbors divided by the total number of neighbors of the node with fewer neighbors. The TOM measure uses the neighbor information instead of just their direct connection strength (adjacency), and is thus a robust measure of interconnectedness. The TOM was converted to a dissimilarity matrix by subtracting all elements from 1 and then used as the input to an average linkage hierarchical clustering function. Modules were identified as well separated branches in the resulting dendrogram. We used dynamic height cut-off 0.995 to cut the clustering tree with minimum module size 40 to reach the 35 proper modules. This module detection approach has led to biologically meaningful modules in several applications [30,32,43-47], but we make no claim that it is optimal.

Tutorials using freely available R software to construct gene co-expression networks and to identify modules are available in the report by Zhang and Horvath [31] and on the internet [48].

## Additional data files

The following additional data are available with the online version of this paper. Additional data file 1 provides The MAGE module of the Chado schema that is pertinent to the representation and storage of microarray data in Celsius.

## Supplementary Material

Additional data file 1 Shown is the MAGE module of the Chado schema that is pertinent to the representation and storage of microarray data in Celsius. Click here for file
