# Supplementary figures and images for: Celsius: a community resource for Affymetrix microarray data
Source: Genome Biol. 2007 Jun 14;8(6):R112. doi: 10.1186/gb-2007-8-6-r112 (PMC2394754; doi:10.1186/gb-2007-8-6-r112)

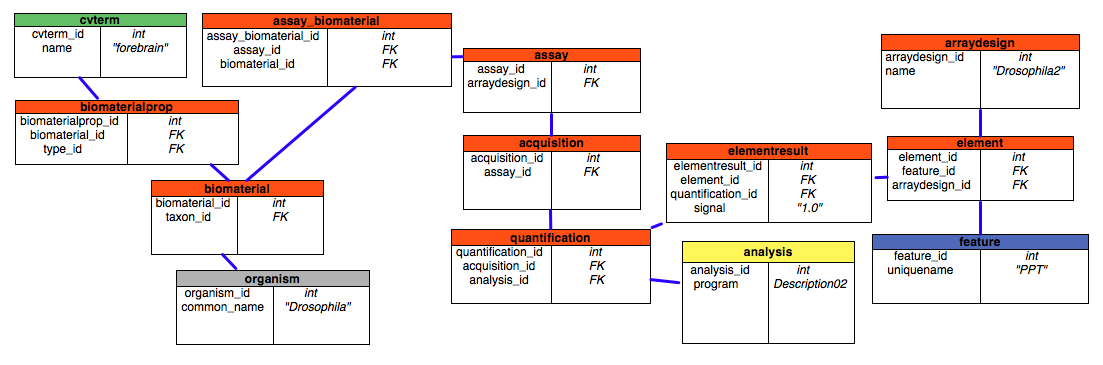

Supplement: Additional data file 1 — Shown is the MAGE module of the Chado schema that is pertinent to the representation and storage of microarray data in Celsius. [file gb-2007-8-6-r112-S1.tiff]
